# Application of auricular therapy in postherpetic neuralgia: a scoping review

**DOI:** 10.3389/fnmol.2026.1790658

**Published:** 2026-05-13

**Authors:** Xiaoyang Wang, Hejia Wan, Xiaohui Liu, Wuyang Tian, Yuxiao Liu, Zilin Zhao, Xin Wang

**Affiliations:** 1 School of Nursing (Nursing School of Smart Healthcare Industry), Henan University of Chinese Medicine, Zhengzhou, Henan, China; 2 Henan Provincial Hospital of Traditional Chinese Medicine, The Second Affiliated Hospital of Henan University of Traditional Chinese Medicine, Zhengzhou, Henan, China; 3 The Second Department of Pediatric, First Affiliated Hospital of Henan University of Chinese Medicine, Zhengzhou, Henan, China

**Keywords:** auriculotherapy, molecular mechanism, pain management, postherpetic neuralgia, scoping review, transcutaneous auricular vagus nerve stimulation

## Abstract

**Objective:**

To conduct a scoping review on the application of auricular therapy in treating postherpetic neuralgia (PHN), comprehensively examining the current clinical status, efficacy, and potential mechanisms of different auricular therapy modalities, while identifying gaps and limitations in existing research to inform further exploration of auricular therapy for PHN.

**Methods:**

Following the Arksey and O’Malley scoping review framework, we searched Chinese full-text journal databases (CNKI), Wanfang Database, VIP Database, SinoMed, PubMed, Web of Science, Cochrane Library, and Embase. The search period spanned from database inception to August 2025. Included studies were analyzed and summarized.

**Results:**

A total of 32 studies were included, with randomized controlled trials being the predominant study type (*n* = 23). Diverse auricular therapy modalities for treating PHN were employed, including auricular seed therapy (bean implantation), auricular acupuncture (fine needles or taper needles), auricular bloodletting, auricular massage, and transcutaneous auricular vagus nerve stimulation (taVNS). Intervention strategies can be categorized into three aspects: point selection, stimulation parameters, and intervention cycles. Core points selected include Shenmen, Subcortical, Sympathetic, and Liver. Stimulation parameters and intervention cycles showed significant heterogeneity, with patch replacement cycles typically ranging from 2 to 7 days and total treatment durations mostly spanning 2 to 4 weeks. Primary outcome measures primarily involved clinical efficacy, pain intensity, and adverse reactions, while a few studies also addressed sleep quality, emotional state, and immune-inflammatory markers.

**Conclusion:**

Auricular therapy represents a significant adjunctive approach for pain management in PHN patients. Existing research faces limitations in intervention protocol standardization, objective validation of mechanisms, and long-term efficacy assessment. Future studies should focus on precise point selection, standardized stimulation parameters, and multidimensional objective outcome measures.

**Systematic review registration:**

DOI: 10.17605/OSF.IO/J4BS2.

## 1 Introduction

Postherpetic neuralgia (PHN) is defined as pain persisting for 1 month or longer after the resolution of the Herpes Zoster (HZ) rash, representing the most common complication of HZ (Yu et al., 2016). According to a recent systematic literature review encompassing 124 studies worldwide (Giannelos et al., 2024), the incidence of PHN following HZ varies considerably across populations, ranging from 2.6 to 46.7% in the general population, with higher rates observed in immunocompromised individuals (3.9–33.8%) and those infected with human immunodeficiency virus (HIV) (6.1–40.2%). PHN is characterized by a protracted course, with pain often persisting for months to years, and in some cases exceeding a decade (Curran et al., 2022; Chinese Medical Doctor Association Dermatologist Branch Herpes Zoster Expert Consensus Working Group and National Clinical Research Center for Dermatologic and Immunologic Diseases, 2022). Beyond causing significant physical distress, it commonly triggers negative emotions such as anxiety and depression, severely impacting social functioning and quality of life. Some patients even develop suicidal tendencies (van Oorschot et al., 2021), making its standardized management a key focus of clinical research (Mbinta et al., 2022).

The primary treatment approaches currently involve local or systemic drug therapy combined with neurointerventional or neuromodulation techniques [techniques (Yu et al., 2016; Lim et al., 2024)]. However, these methods remain limited in terms of safety, accessibility, and cost-effectiveness, with some patients still experiencing inadequate pain control (Peng and Min, 2025; Kim and Kim, 2026). There is an urgent need to explore safe, effective, and economical complementary treatment options. Auricular therapy, as a traditional medical auxiliary therapy, has the advantages of simplicity, convenience, effectiveness and economy. It has been widely applied in the treatment of tumor symptoms, pain control and tumor-related complications at present (Li et al., 2026; Li F. et al., 2025; Dai et al., 2025; Wang et al., 2021). Multiple studies (Peng and Min, 2025; Mueller, 2023; Li and Wang, 2025) have confirmed its pain-relieving effects on neuropathic pain. However, current intervention protocols, efficacy assessments, and outcome measures for auricular therapy in PHN treatment vary significantly, lacking standardized approaches that hinder its clinical dissemination and high-quality application. Therefore, this study systematically reviews the current clinical application of auricular therapy for PHN based on the scope review methodology framework proposed by Arksey and O’Malley (Arksey and O'malley, 2005). It identifies research gaps and deficiencies to provide a foundation for establishing standardized intervention protocols and conducting high-quality research in the future.

## 2 Materials and methods

### 2.1 Define the research question

Scoping review, as a systematic method for literature synthesis, has been widely applied in oncology, nursing, critical care medicine, and complementary and alternative medicine (Li et al., 2026; Li F. et al., 2025; Guo et al., 2026; Guo et al., 2025). This scoping review was conducted in accordance with the methodological framework proposed by Arksey and O’Malley. The primary objective is to map the existing evidence on auricular therapy for postherpetic neuralgia (PHN) and to identify knowledge gaps to inform future research. According to the Population–Concept–Context (PCC) framework proposed by Arksey and O’Malley, we pose the following research questions: What types of auricular therapy are used to treat PHN? What are the intervention protocols for different types of auricular therapy in treating PHN (including acupoint selection, stimulation parameters, intervention cycles, and main outcome measures)? What types of efficacy outcomes have been reported in the existing literature on auricular therapy for PHN? What are the potential mechanisms of action for auricular therapy in treating PHN? What are the current limitations of auricular therapy for PHN, and what might be the future research directions?

### 2.2 Search strategy

This review systematically searched nine databases, including PubMed, Web of Science, Cochrane Library, Scopus, CINAHL, as well as Chinese journal databases CNKI, Wanfang, VIP, and SinoMed. The search period spanned from the establishment of each database up to December 2025, employing a combination of subject headings and free-text terms. The search strategy for English databases is detailed in Table 1 (e.g., PubMed).

**Table 1:** Literature retrieval strategy.

Database type	Search query	
Taking PubMed as an example	#3	#1 AND #2
	1#	((“acupunctural”[All Fields] OR “Acupuncture”[MeSH Terms] OR “Acupuncture”[All Fields] OR “acupuncture therapy”[MeSH Terms] OR (“Acupuncture”[All Fields] AND “therapy”[All Fields]) OR “acupuncture therapy”[All Fields] OR “acupuncture s”[All Fields] OR “acupunctured”[All Fields] OR “Acupunctures”[All Fields] OR “acupuncturing”[All Fields]) AND “Ear”[Title/Abstract]) OR ((“Ear”[MeSH Terms] OR “Ear”[All Fields]) AND “Acupunctures”[Title/Abstract]) OR “acupuncture auricular”[Title/Abstract] OR ((“acupunctural”[All Fields] OR “Acupuncture”[MeSH Terms] OR “Acupuncture”[All Fields] OR “acupuncture therapy”[MeSH Terms] OR (“Acupuncture”[All Fields] AND “therapy”[All Fields]) OR “acupuncture therapy”[All Fields] OR “acupuncture s”[All Fields] OR “acupunctured”[All Fields] OR “Acupunctures”[All Fields] OR “acupuncturing”[All Fields]) AND “Auricular”[Title/Abstract]) OR “auricular acupunctures”[Title/Abstract] OR “auricular acupuncture”[Title/Abstract] OR “ear acupuncture”[Title/Abstract] OR “acupuncture, ear”[MeSH Terms]
	2#	((Acupunctures, Ear[Title/Abstract] OR Ear Acupunctures[Title/Abstract] OR Acupuncture, Auricular[Title/Abstract] OR Acupunctures, Auricular[Title/Abstract] OR Auricular Acupunctures[Title/Abstract] OR Auricular Acupuncture[Title/Abstract] OR Ear Acupuncture[Title/Abstract]) OR (“Acupuncture, Ear”[Mesh])) OR ((Auriculotherapies[Title/Abstract]) OR (“Auriculotherapy”[Mesh]))
	3#	(((((((((((auricular vagus nerve[Title/Abstract]) OR (aVN[Title/Abstract])) OR (low-level tragus stimulation[Title/Abstract])) OR (LLTS[Title/Abstract])) OR (auricular acupuncture points[Title/Abstract])) OR (AAPs[Title/Abstract])) OR (transcutaneous auricular vagus nerve stimulation[Title/Abstract])) OR (taVNS[Title/Abstract])) OR (transcutaneous electrical stimulation AAPs[Title/Abstract])) OR (transcutaneous electrical stimulation auricular acupuncture points[Title/Abstract])) OR (transcutaneous electrical stimulation AAPs distribution VN[Title/Abstract])) OR (transcutaneous electrical stimulation auricular acupuncture points distribution of vagus nerve[Title/Abstract])
	#4	#1 OR #2 OR #3
	#5	“postherpetic neuralgia”[Title/Abstract] OR “postherpetic neuralgia”[Title/Abstract] OR “neuralgia, postherpetic”[MeSH Terms]
	#6	#4 AND #5

### 2.3 Inclusion criteria and exclusion criteria

Inclusion and exclusion criteria were established in accordance with the principles of PCC.

Inclusion criteria:

(1) Population were patients with a confirmed diagnosis of PHN. PHN was defined as persistent pain lasting one month or longer following the resolution of the herpes zoster rash.
(2) Concept: Various auricular therapy modalities, including auricular acupressure, auricular acupuncture, auricular bloodletting, and taVNS, with clearly defined outcome assessment tools.
(3) Context refers to the application of auricular therapy in patients with postherpetic neuralgia, encompassing original studies such as randomized controlled trials, quasi-experimental studies, mixed-method studies, and qualitative research, regardless of study location.

Exclusion criteria:

(1) Failure to describe auricular therapy interventions in detail;
(2) Studies unrelated to PHN pain;
(3) Literature not in Chinese or English;
(4) Full text unavailable;
(5) Duplicate publications.

### 2.4 Study selection and data extraction

Initially retrieved literature was imported into NoteExpress software for preliminary deduplication processing, followed by manual deduplication through setting duplicate detection fields. Two researchers trained in evidence-based nursing rigorously applied inclusion and exclusion criteria. They conducted independent initial screening by reviewing titles and abstracts, followed by secondary screening based on full-text content to exclude irrelevant studies. Disagreements during the literature screening process were resolved through discussion with the third researcher. Use Microsoft Excel to extract literature information, including authors, country, date, study design, study purpose, sample size, intervention protocols for the experimental and control groups, Outcome measures.

### 2.5 Data analysis and synthesis

Extracted data were analyzed using a descriptive categorization approach. Based on the research objectives, the information was grouped into the following categories:

(1) types of auricular therapy.
(2) intervention protocols (acupoint selection, stimulation parameters, intervention cycles)
(3) reported outcome measures.
(4) potential mechanisms of action.
(5) limitations of existing studies.

A descriptive summary approach was employed to present the findings, with results organized in tables and accompanying narrative synthesis.

## 3 Results

### 3.1 Study selection

**Figure 1:**
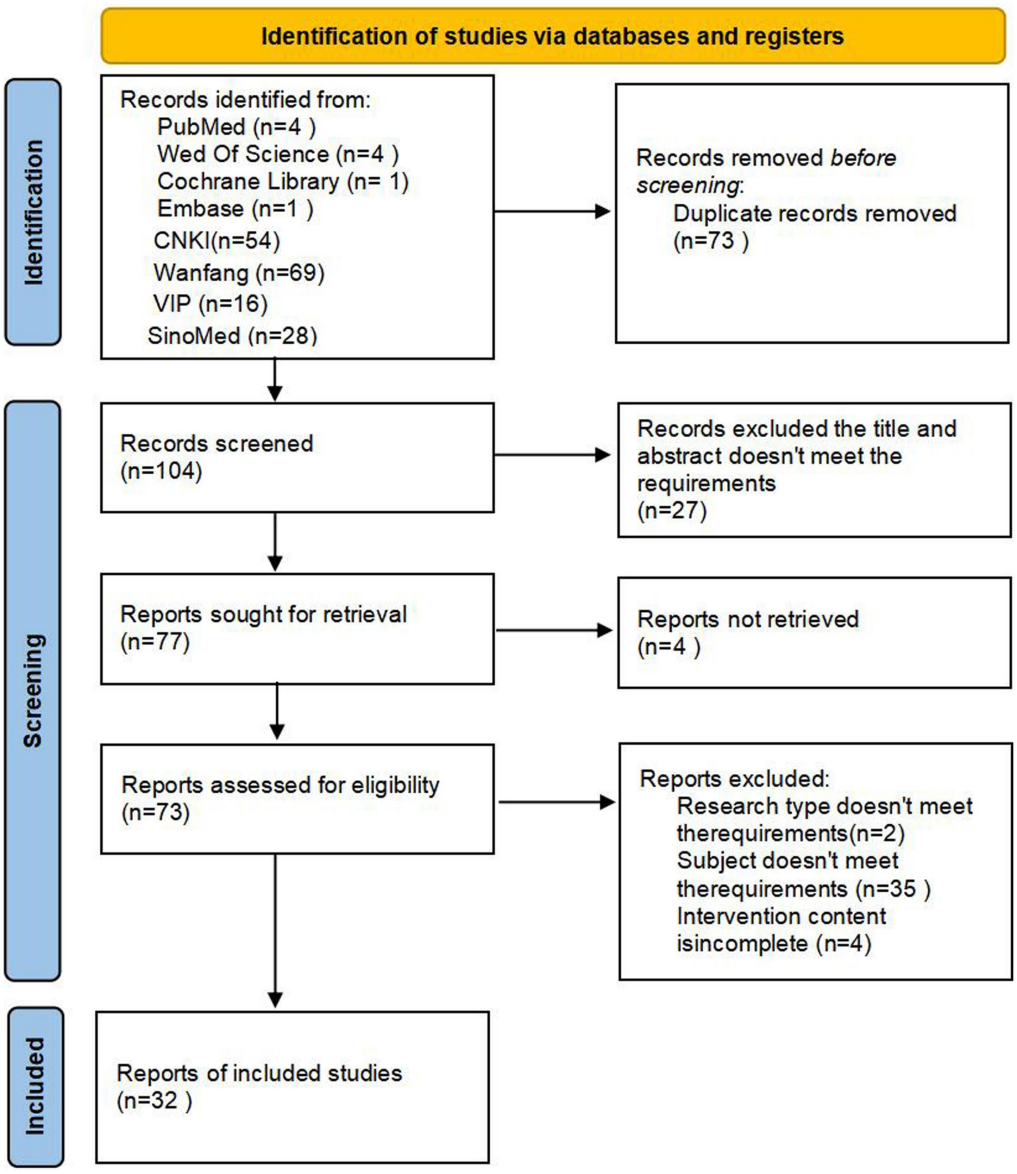
PRISMA 2020 flow diagram of study selection.

A total of 179 records were initially identified. Following the removal of 73 duplicates, the titles and abstracts of 106 records were screened, resulting in the exclusion of 27. After full-text assessment of the remaining 79 articles, 45 were excluded, leaving 32 studies eligible for inclusion in the review. The selection process is illustrated in the PRISMA flow diagram (Figure 1).

### 3.2 Study characteristics

Among the 32 documents, 31 were in Chinese (Liu et al., 2025; Feng et al., 2025; Wen et al., 2024; Wang et al., 2024; Zhao et al., 2024a; Yu, 2024; Le et al., 2024; Xu, 2024; Lu, 2023; Wang, 2023; Ye and Zhan, 2023; Zhao et al., 2022; Yu et al., 2022; Chen, 2021; Chen et al., 2021; Chen et al., 2020; Li J. et al., 2020; Yu et al., 2020; Wang et al., 2019; Wang, 2015; Ruan et al., 2014; Liu et al., 2014; Xu et al., 2013; Yang, 2012; Cai, 2011; Bao et al., 2010; Chen et al., 2009; Wu et al., 2007; Ding, 2007; Tang et al., 2005; Liu, 2004) and 1 was in English (Wang, 2007). Thirty-one studies were from China (Liu et al., 2025; Feng et al., 2025; Wen et al., 2024; Wang et al., 2024; Zhao et al., 2024a; Yu, 2024; Le et al., 2024; Xu, 2024; Lu, 2023; Wang, 2023; Ye and Zhan, 2023; Zhao et al., 2022; Yu et al., 2022; Chen, 2021; Chen et al., 2021; Chen et al., 2020; Li J. et al., 2020; Yu et al., 2020; Wang et al., 2019; Wang, 2015; Ruan et al., 2014; Liu et al., 2014; Xu et al., 2013; Yang, 2012; Cai, 2011; Bao et al., 2010; Chen et al., 2009; Wu et al., 2007; Ding, 2007; Tang et al., 2005; Liu, 2004), and one was from the United States (Wang, 2007). Publication dates ranged from 2004 to 2025. Study types included 23 randomized controlled trials (Liu et al., 2025; Feng et al., 2025; Wen et al., 2024; Yu, 2024; Le et al., 2024; Lu, 2023; Wang, 2023; Ye and Zhan, 2023; Zhao et al., 2022; Yu et al., 2022; Chen, 2021; Chen et al., 2020; Li J. et al., 2020; Yu et al., 2020; Wang, 2015; Ruan et al., 2014; Liu et al., 2014; Yang, 2012; Bao et al., 2010; Chen et al., 2009; Wu et al., 2007; Tang et al., 2005; Liu, 2004),5 quasi-experimental studies (Zhao et al., 2024a; Xu, 2024; Xu et al., 2013; Cai, 2011; Ding, 2007), 1 observational study (Wang et al., 2024), and 3 case reports (Chen et al., 2021; Wang et al., 2019; Wang, 2007). The basic characteristics of the included literature are shown in Table 2.

**Table 2:** Characteristics of the included studies (*n* = 32).

Authors	Country	Date	Study design	Sample size (T/C)	Intervention protocols for test group					Intervention protocols for the control group	Outcome measures	Potential mechanisms reported	Limitations reported
					Intervention measures	Ear acupuncture point	Pressure frequency and duration	Intensity and replacement cycle	Total treatment duration				
Liu et al. (2025)	China	2025	Randomized Controlled Trial	53/53	Peony and Licorice Decoction with Additional Ingredients combined with Auricular Acupuncture Therapy	Shenmen, Sympathetic, Kidney, Spleen, Subcortex point	Press 3 to 5 times daily, approximately 90 s each time.	Single-ear taping for 7 days constitutes one treatment course.	4 weeks	Oral Gabapentin Capsules	①②③⑤⑧	The combination therapy modulated pain-related neuropeptides, T lymphocyte subsets, and the PI3K/AKT/mTOR signaling pathway.	Not Reported
Feng et al. (2025)	China	2025	Randomized Controlled Trial	20/20	Auricular Acupuncture Therapy	Heart, Stomach, Upper Screen Point, Liver-Gallbladder, Adrenal, Wind Creek	Press 4 times daily, each session lasting 3 to 5 min.	Not Reported	14 days	Standard treatment	①②⑧	Regulates meridians and viscera functions; exerts sedative, analgesic, and sleep-promoting effects.	Not Reported
Wen et al. (2024)	China	2024	Randomized Controlled Trial	44/43	Auricular Acupuncture Therapy	Liver, Shenmen, Subcortical, Lung, Adrenal	Press 4–6 times daily, each time for 1 min.	Change every 4 days	2 weeks.	Standard treatment (Apply acyclovir ointment to the affected area. Administer vitamin B12 and vitamin B11 via intramuscular injection.)	①②⑤⑦	Regulates immune function (CD4+/CD8 + balance), reduces inflammatory cytokines (TNF-α, IL-6, IL-8), blocks calcium channels, and promotes neurotrophic factor secretion.	Not Reported
Wang et al. (2024)	China	2024	Case–Control Study	30/41	Qingpeng Ointment Applied Topically Combined with Auricular Acupuncture	Shenmen, Lung, Subcortical, Liver	Each needle remains in place for 24 h.	Alternating ears	30 days	Standard treatment combined with Qingpeng Ointment Applied Topically	①②③④⑤⑥⑨	Reduces inflammatory markers (IL-6, TNF-α, CRP).	Retrospective, single-center study; potential selection bias; small sample size; no blank control group.
Zhao et al. (2024a)	China	2024	Pre-Post Study	82	Auricular Acupuncture Therapy combined with Acupoint Injection	Shenmen, Sympathetic, Subcortical, Spleen, Liver, Adrenal	Press each point for 1 min, 4 times daily.	Alternate between ears every 3 days.	60 days	—	②③④	Regulates meridians, promotes blood circulation, relieves pain; acupoint injection unblocks meridians, removes blood stasis, and balances yin and yang.	Short observation period; further prospective studies with longer follow-up and larger sample size are needed.
Yu (2024)	China	2024	Randomized Controlled Trial	40/40	Auricular Acupuncture Therapy combined with Oral Administration of Pregabalin Capsules	Endocrine, Sympathetic, Subcortical, Shenmen; Liver, Eye, Lung, Cheek Area, Heart, Large Intestine, Occipital	Massage 5–7 times daily.	Alternate ears every 2 days.	7 days	Standard treatment and care, oral premedication with pregabalin capsules	①②⑦⑧	Auricular acupressure may promote endogenous endorphin release, act on enkephalin receptors, and modulate central pain signal interaction.	Not Reported
Le et al. (2024)	China	2024	Randomized Controlled Trial	47/48	Combined Bloodletting and Cupping with Auricular Acupuncture	Heart, Shenmen, Endocrine, Subcortical, Sympathetic, Thoracic Spine, Lumbar Spine	Leave needles in place for 72 h. Apply pressure 3–5 times daily, 1 min per acupoint each time.	Alternating ears	4 weeks.	Oral pregabalin capsules	①②③⑤⑥⑧⑨	Auricular thumbtack needle improves immune function (increases IgG) and reduces inflammatory cytokines (IL-6, CRP).	Lack of long-term follow-up; future studies should include follow-up to assess long-term efficacy.
Xu (2024)	China	2024	Pre-Post Study	40/40	Auricular Acupuncture Therapy combined with Wrist-ankle acupuncture	Ear Tip, Wind Stream, Pancreas-Gallbladder, Lung, Liver, Endocrine, Adrenal; For severe pain, add Spirit Gate and Occipital; For insomnia, add Chin Front.	Press each point for 2 min per session, 60 times per minute.	Alternating ears	2 weeks.	Conventional drug therapy combined with Auricular Acupuncture Therapy	①②⑤⑦⑧⑨	Regulates meridians, coordinates zang-fu organs, promotes blood circulation, removes blood stasis, reduces swelling, and relieves pain (TCM theory).	Not Reported
Lu (2023)	China	2023	Randomized Controlled Trial	35/35	Auricular Acupuncture Therapy combined with Infrared radiation	Shenmen, Sympathetic, Adrenal, Subcortical; Liver, Pancreatic-Gallbladder, Spleen; additional points may be selected based on the location of pain, such as Shoulder, Chest, Abdomen, Neck, etc.	Press each point 3 times daily for 1 to 2 min per session.	Not Reported	14 days	Conventional drug therapy	①②③⑥	Regulates cerebral cortex excitation, provides analgesia and anti-inflammation.	Not Reported
Wang (2023)	China	2023	Randomized Controlled Trial	14/14	Auricular Acupuncture	Liver, Ear Tip, Shenmen, Spleen, and Ear Points Corresponding to Skin Lesions on the Lower Back	Once daily	Pinch and lift the earlobe for 4 min per session; Massage the helix until it becomes flushed and warm; Pinch and rub the ear tip 20 times.	1 week.	Standard Treatment	①②③④	Auricular massage may stimulate endogenous production of analgesic and sedative substances, regulate nervous system function, and enhance immune function.	Not Reported
Ye and Zhan (2023)	China	2022	Randomized Controlled Trial	30/30	Auricular Acupuncture Therapy combined with Topical application of traditional Chinese medicine	Stomach, Ear Tip, Shenmen, Endocrine, Adrenal	Press for 1–2 min, 3–4 times daily.	Alternate ears every 3–4 days.	4 weeks.	Conventional medical treatment	①②④⑨	Auricular acupressure may stimulate nerve endings to release endorphin-like substances, increase pain threshold, reduce pain sensitivity, regulate nerve function, and promote nerve repair.	Not reported.
Zhao et al. (2022)	China	2022	Randomized Controlled Trial	80/80	Auricular Acupuncture Therapy combined with Acupressure	Shenmen, Sympathetic, Subcortical; Spleen, Liver, Adrenal Glands	Press each point for 1 min (with 10-s intervals) during each of the three daily meals and before bedtime.	Alternate ears every 3–4 days.	30 days	Standard Treatment and Care	①②③④⑦	Auricular acupressure may activate blood, relieve pain, and regulate nerve function based on TCM meridian and zang-fu organ theory. Acupoint massage may relax meridians, activate collaterals, promote qi and blood circulation, and relieve muscle tension.	Not reported.
Yu et al. (2022)	China	2022	Randomized Controlled Trial	120/120	Bloodletting behind the ear combined with Auricular Acupuncture Therapy	Shenmen, Endocrine, Subcortical, Liver, Pancreaticobiliary	Press 3 times daily, 5 min each time.	Alternate ears every 3 days.	30 days	Oral Lofen-Codeine Extended-Release Tablets	①②③⑤⑧	Auricle bloodletting removes blood stasis, clears heat, reduces swelling, relieves pain, detoxifies, and may repair cells and enhance immunity. Auricular point pressing stimulates ear acupoints, regulates nerve function, reduces inflammation, and relieves pain.	Low patient compliance; long-term efficacy needs further study.
Chen (2021)	China	2021	Randomized Controlled Trial	45/45	Bloodletting behind the ear combined with Electroacupuncture, moxibustion	Adrenal gland, lung, subcortical region, Shenmen point, liver	Press 4–5 times daily	Alternate ears every 3–5 days.	1 month	Conventional drug therapy	①②⑦⑧	Calms mind and relieves pain; harmonizes zang-fu organs and balances yin and yang.	Not reported.
Chen et al. (2021)	China	2021	Case Report	1	The “Six Points of the Middle Town” acupuncture therapy combines auricular acupuncture with body acupoints.	Shenmen, Heart, Lung;	3 times a week	Alternating ears	2 weeks	—	②③⑥	Calms mind, relieves pain, regulates qi and blood, clears liver heat, unblocks meridians (TCM theory)	Case report; low level of evidence.
Chen et al. (2020)	China	2020	Randomized Controlled Trial	10/10	taVNS combined with oral pregabalin	Concha region (distribution area of the auricular branch of the vagus nerve)	Twice daily (9–10 a.m. and 8–9 p.m.), 30 min each session	Using the TENS-200A stimulator with a pulse frequency of 20 Hz (alternating density waveform), pulse width ≤1 mA, and current starting at 1 mA, gradually increasing to the patient’s tolerable and pain-free level.	1 week	Pseudo-stimulation combined with oral pregabalin	②⑤⑧	taVNS may inhibit CD4 + T-cell activation (reduced CD71 expression) and downregulate pro-inflammatory cytokines (TNF-α, IL-1β).	Small sample size; short treatment duration; no long-term follow-up.
Li J. et al. (2020)	China	2020	Randomized Controlled Trial	42/38	Auricular Acupuncture Therapy combined with a formula to tonify qi and nourish yin	Shenmen, Upper Screen Point, Endocrine, Liver, Subcortical; For upper limb pain, add Finger and Shoulder points; For lower limb pain, add Toe and Buttock points; For facial pain, add Cheek and Occipital points.	Press 3 times daily, 2 min each time.	Replace every 2 weeks	3 weeks	Conventional medical treatment combined with a formula to tonify qi and nourish yin	①②⑤⑧	Sends electrical signals to the CNS via vagus nerve; reduces serum CRP; clears heat, unblocks meridians, relieves pain, enhances immunity (TCM).	Not reported.
Yu et al. (2020)	China	2020	Randomized Controlled Trial	49/48	Combined drug therapy with auricular bloodletting	The bloodletting site on the back of the ear is the superficial capillary veins located in the upper third of the ear, near the helix.	Once a week	Alternate ears every 2 days.	4 weeks	Oral medication therapy	①②③⑧	It improves microcirculation, promotes tissue repair and regeneration, enhances nonspecific immunity, regulates neuroendocrine and immune functions, and reduces pain by stimulating the vagus nerve in the ear to increase parasympathetic tone.	Not reported.
Wang et al. (2019)	China	2019	Case Report	1	Zhuang’s “Spirit Regulation Acupuncture Method” Combined with Auricular Acupuncture	Heart, Subcortical, Chest, Shenmen, Spleen, Sympathetic, Ear Center	Once daily, leave the needle in for 30 min.	Alternate ears every 3 days.	Once daily	—	①②③⑥⑨	Regulates mental state, calms mind, improves sleep, unblocks meridians; strong stimulation suppresses pathological cortical excitation.	Not reported.
Wang (2015)	China	2015	Randomized Controlled Trial	38/28	Combination of Traditional Chinese Medicine and Auricular Acupuncture	Shangpingjian, Liver, Shenmen. For upper limb pain, add: fingers, shoulder. For lower limb pain, add: toes, upper ear back. For waist and abdominal pain, add: abdomen, upper ear root. For head and facial pain, add: cheeks, occipital region.	Press 3–4 times daily, each time for 3–5 min.	Replace once a week	2 weeks	Conventional drug therapy	①②⑧	Clears heat, removes toxins, unblocks meridians; produces direct analgesic effects and enhances regulatory functions.	Not reported.
Ruan et al. (2014)	China	2014	Randomized Controlled Trial	55/55	Electroacupuncture combined with auricular acupressure	Shenmen, Endocrine, Subcortical, Spleen, Lung, and corresponding disease-area auricular points.	Press 3 times daily, 3 min each time.	Alternate ears every 3 days.	3–4 weeks	Oral medication therapy	①②③	Calms mind, relieves pain, strengthens spleen, nourishes blood, regulates immunity.	Not reported.
Liu et al. (2014)	China	2014	Randomized Controlled Trial	61/61	Fire Needle Combined with Ear Acupuncture Point Pressure	Lung, Shenmen, Liver, Gallbladder, and corresponding ear points at the site of pain.	Every 2 days	Rest for 2 days between each treatment cycle.	16 days (including 2 rest days)	Electroacupuncture	①②⑧	Not reported.	Not reported.
Xu et al. (2013)	China	2013	Pre-Post Study	26	Stellate Ganglion Block Combined with Auricular Acupuncture	Shenmen, Endocrine, Subcortical, Liver, Gallbladder, Lung	Press each point 3 to 5 times daily, holding for 30 to 60 s per point each time.	Alternate ears every 3–7 days.	Not Reported	—	①②⑦	Unblocks meridians, promotes qi and blood circulation, regulates physiological functions of zang-fu organs, improves organ function via neural pathways.	Not reported.
Yang (2012)	China	2012	Randomized Controlled Trial	30/26	Combined auricular acupuncture and auricular acupressure	For auricular acupuncture, select the Stomach point. For auricular pressure therapy, select the Shenmen, Endocrine, Adrenal, and corresponding disease-area auricular points.	Ear acupuncture: once daily, retain needles for 15 min; Ear acupressure patches: applied after needling, press each point 5–6 times daily for 1 min per point.	Alternate ears every 5 days.	30 days	Standard treatment	①②⑧⑨	Regulates overall body functions based on biological holography theory; activates qi and blood, unblocks meridians, relieves pain, enhances immune regulation.	Not reported.
Cai (2011)	China	2011	Pre-Post Study	20/20	Auricular Acupuncture Combined with Spinal Paravertebral Acupuncture	Liver, spleen, Shenmen, ear tip, and Ear Points Corresponding to Skin Lesions on the Lower Back	Pull the earlobe for 3–5 min per session. Massage the auricle until it becomes flushed and warm. Pull the ear tip 15–20 times per session.	Each treatment course lasts 5 to 7 days.	18 ~ 40 days	Standard treatment	①②	Clears heat, removes toxins, promotes qi circulation, resolves blood stasis, unblocks meridians; regulates neuro-endocrine-immune function, promotes tissue repair.	Not reported.
Bao et al. (2010)	China	2010	Randomized Controlled Trial	33/32	Acupuncture at Six Points in the Middle of the Town (Including Auricular Acupuncture) Combined with Plum Blossom Needle Meridian Stimulation and Cupping	Shenmen, Heart, Lung	Not Reported	Alternate ears every 2 days.	30 days	Oral medication therapy	①②④⑧	Calms mind, relieves pain, promotes blood circulation, unblocks meridians, regulates lung qi (TCM: “all pains and itching sores belong to the heart”).	Not reported.
Chen et al. (2009)	China	2009	Randomized Controlled Trial	35/35	Laser irradiation combined with auricular acupressure	Liver, Gallbladder, Endocrine, Subcortical, Shenmen,and corresponding disease-area auricular points.	Press 4–6 times daily.	Select 3–4 acupoints each time, Alternate ears every 3 days.	20 days	Oral medication therapy	①②⑦⑧	Auricular point pressing may regulate cerebral cortex excitation and inhibition, modulate autonomic nerve and vasomotor function, and provide sedation and analgesia.	Small sample size; short follow-up period.
Wu et al. (2007)	China	2007	Randomized Controlled Trial	60/56	Auricular Acupuncture Combined with Acupoint Injection Therapy	Shenmen, Endocrine, Subcortical, Liver, Gallbladder, Lung, and corresponding disease-area auricular points.	Press 4 to 6 times daily, for 1 min each time.	Alternate ears every 3 days.	21 days	Oral medication therapy	①②⑧	Auricular point pressing may calm the mind, relieve pain, soothe the liver and gallbladder, and regulate immune function based on the connection between ear meridians and zang-fu organs.	Not reported.
Ding (2007)	China	2007	Pre-Post Study	26	Auricular Acupuncture Combined with Bloodletting and Cupping Therapy and Microwave Heat Therapy	Ashi point (the reflex point on the ear corresponding to the painful area)	Self-massage each point 4–5 times daily, holding each point for 2–3 min.	Not Reported	7 days	—	①②⑧	Not reported.	Not reported.
Wang (2007)	United States	2007	Case Report	1	Auricular Acupuncture Combined with Body Acupuncture and TAES	Right ear, mandible, face, inner ear, and Shenmen.	Self-massage every night before bed	Not Reported	Not Reported	—	②⑥⑧⑨	Auricular acupressure and auricular acupuncture may regulate the flow of “Qi” in meridians, trigger the release of endogenous opioids (e.g., via different electroacupuncture frequencies), increase pain threshold, and inhibit C-fiber afferent input, thereby providing analgesia.	Case report; low level of evidence.
Tang et al. (2005)	China	2005	Randomized Controlled Trial	50/40	Triple Meridian Therapy (Ear-Back Bloodletting, Auricular Acupuncture, Acupoint Implantation)	Bilateral ear sensitivity points (pain, numbness, and distension points)	Press 100 to 200 times daily.	Once every 3 days	4–6 weeks	Oral medication therapy	①②⑧	Auricular bloodletting and auricular point pressing may unblock meridians, remove blood stasis, clear heat, relieve pain, regulate neuro-humoral function, improve microcirculation, enhance cellular metabolism and immune function, and promote nerve repair.	Not reported.
Liu (2004)	China	2004	Randomized Controlled Trial	21/23	Combined auricular acupuncture and ear tip bloodletting	Shenmen, Gallbladder, Liver, Lung, Subcortical, Adrenal. For itching, add Fengxi and Diaphragm points; for severe dampness and numbness, add Spleen points.	Once every 3 days	Not Reported	15 days	Oral Gentiana Liver-Draining Decoction with Modifications	①②	Not reported.	Not reported.

TCM, Traditional Chinese Medicine; ① Clinical efficacy; ② Pain intensity; ③ Sleep quality; ④ Quality of Life; ⑤ Immune Function and Inflammation-Related Markers; ⑥ Emotional and Psychological State; ⑦ Recurrence rate; ⑧ Incidence of adverse reactions; ⑨ Other: Nursing satisfaction, Traditional Chinese Medicine symptom scores, time to onset of analgesia, changes in therapeutic efficacy, etc.

### 3.3 Types of auricular therapy

The 32 included studies encompassed five major categories of auricular therapy, which can be broadly classified into traditional, modern, and composite approaches. These include auricular seed therapy (*n* = 19) (Liu et al., 2025; Feng et al., 2025; Wen et al., 2024; Zhao et al., 2024a; Yu, 2024; Xu, 2024; Lu, 2023; Ye and Zhan, 2023; Zhao et al., 2022; Chen, 2021; Li J. et al., 2020; Wang, 2015; Ruan et al., 2014; Liu et al., 2014; Xu et al., 2013; Chen et al., 2009; Wu et al., 2007; Ding, 2007; Wang, 2007), auricular acupuncture (fine needles or tapered needles) (*n* = 7) (Wang et al., 2024; Le et al., 2024; Chen et al., 2021; Wang et al., 2019; Yang, 2012; Bao et al., 2010; Liu, 2004), auricular bloodletting (*n* = 3) (Yu et al., 2022; Yu et al., 2020; Tang et al., 2005), auricular acupressure (*n* = 2) (Wang, 2023; Cai, 2011), and transcutaneous auricular vagus nerve stimulation (taVNS) (*n* = 1) (Chen et al., 2020). Bloodletting at the ear lobe or ear tip typically involves puncturing the auricle with a three-edged needle to release a small amount of blood. This method is primarily used to treat acute, solid, and heat-related conditions, as well as dermatological disorders. Ear massage involves stimulating acupoints on the auricle using a probe or fingers. Its non-invasive nature and ease of application make it suitable for adjunctive treatment and daily health maintenance across various conditions. A few studies have incorporated modern techniques like transcutaneous vagus nerve stimulation (tVNS) (Chen et al., 2020), which electrically stimulates the auricular branch of the vagus nerve in the concha region, reflecting the trend toward precision neural modulation in auricular therapy. Furthermore, combined approaches integrating different auricular therapies—such as auricular acupuncture with auricular plaster application, auricular plaster application with bloodletting and cupping, or auricular bloodletting combined with auricular plaster application—have been employed in clinical practice and research for PHN pain management to enhance analgesic effects.

### 3.4 Auricular therapy intervention protocol

#### 3.4.1 Principles for selecting acupoints

In the included studies, auricular point selection adhered to the principle of integrating pattern differentiation with empirical point selection. High-frequency selection results identified core auricular points for treating PHN. For analgesia and neural regulation: Shenmen (Liu et al., 2025; Wen et al., 2024; Wang et al., 2024; Zhao et al., 2024a; Yu, 2024; Le et al., 2024; Xu, 2024; Lu, 2023; Wang, 2023; Ye and Zhan, 2023; Zhao et al., 2022; Yu et al., 2022; Chen, 2021; Chen et al., 2021; Chen et al., 2020; Li J. et al., 2020; Yu et al., 2020; Wang et al., 2019; Wang, 2015; Ruan et al., 2014; Liu et al., 2014; Xu et al., 2013; Yang, 2012; Cai, 2011; Bao et al., 2010; Chen et al., 2009; Wu et al., 2007; Wang, 2007; Liu, 2004), Subcortical (Liu et al., 2025; Wen et al., 2024; Wang et al., 2024; Zhao et al., 2024a; Yu, 2024; Le et al., 2024; Lu, 2023; Zhao et al., 2022; Yu et al., 2022; Chen, 2021; Li J. et al., 2020; Yu et al., 2020; Wang et al., 2019; Ruan et al., 2014; Xu et al., 2013; Chen et al., 2009; Wu et al., 2007; Liu, 2004); Sympathetic (Liu et al., 2025; Zhao et al., 2024a; Yu, 2024; Le et al., 2024; Lu, 2023; Zhao et al., 2022; Wang et al., 2019); anti-inflammatory and immunomodulatory: Adrenal (Feng et al., 2025; Wen et al., 2024; Zhao et al., 2024a; Lu, 2023; Ye and Zhan, 2023; Zhao et al., 2022; Chen, 2021; Liu, 2004), Endocrine (Yu, 2024; Le et al., 2024; Xu, 2024; Ye and Zhan, 2023; Yu et al., 2022; Li J. et al., 2020; Yu et al., 2020; Ruan et al., 2014; Xu et al., 2013; Yang, 2012; Chen et al., 2009; Wu et al., 2007); TCM Pattern Differentiation Categories: Liver (Feng et al., 2025; Wen et al., 2024; Wang et al., 2024; Zhao et al., 2024a; Yu, 2024; Xu, 2024; Lu, 2023; Wang, 2023; Zhao et al., 2022; Yu et al., 2022; Chen, 2021; Li J. et al., 2020; Yu et al., 2020; Wang, 2015; Liu et al., 2014; Xu et al., 2013; Cai, 2011; Chen et al., 2009; Wu et al., 2007; Liu, 2004), Lung (Wen et al., 2024; Wang et al., 2024; Yu, 2024; Xu, 2024; Chen, 2021; Chen et al., 2021; Xu et al., 2013; Bao et al., 2010; Wu et al., 2007; Liu, 2004), Spleen (Liu et al., 2025; Zhao et al., 2024a; Lu, 2023; Wang, 2023; Zhao et al., 2022; Wang et al., 2019; Ruan et al., 2014; Liu et al., 2014); Local Correspondence Category: Ear points selected based on pain location (Lu, 2023; Wang, 2023; Li J. et al., 2020; Wang, 2015; Ruan et al., 2014; Liu et al., 2014; Yang, 2012; Cai, 2011; Chen et al., 2009; Wu et al., 2007; Ding, 2007; Wang, 2007; Liu, 2004); Additionally, one study on transcutaneous vagus nerve stimulation selected the auricular concha (area of vagus nerve auricular branch distribution) (Chen et al., 2020).

#### 3.4.2 Stimulation parameters and intervention cycle

All 32 included studies described intervention protocols for auricular therapy. To characterize the heterogeneity in stimulation protocols, we extracted data across four dimensions: frequency, duration per session, intensity, and replacement/retention cycle.

(1) Frequency of stimulation

For auricular acupressure, daily application frequency typically ranged from 3 to 5 sessions per day, with some studies reporting 4–6 times daily (Wen et al., 2024) or 5–7 times daily (Yu, 2024). For auricular acupuncture (filiform or press needles), needles were typically retained for 24 to 72 h (Wang et al., 2024; Le et al., 2024), often using alternating bilateral ears. For taVNS, stimulation was administered twice daily (Chen et al., 2020).

(2) Duration per session

For auricular acupressure, each point was stimulated for 1 to 5 min per session. Specific durations included approximately 90 s (Liu et al., 2025), 1 min (Wen et al., 2024; Zhao et al., 2024a; Le et al., 2024; Lu, 2023; Zhao et al., 2022; Wu et al., 2007), 2 min (Xu, 2024; Li J. et al., 2020), 3 min (Feng et al., 2025; Yu et al., 2022; Ruan et al., 2014), and 3–5 min (Wang, 2015; Cai, 2011). For taVNS, each session lasted 30 min (Chen et al., 2020).

(3) Intensity

For auricular acupressure, pressure intensity was adjusted to elicit sensations of fullness, warmth, or numbness in patients (Liu et al., 2025; Lu, 2023; Zhao et al., 2022; Wang, 2015). For taVNS, stimulation frequency predominantly employed 20 Hz sparse-dense waves, with current intensity starting at 1 mA and gradually increasing to the patient’s tolerable and pain-free level (Chen et al., 2020).

(4) Replacement/retention cycle

For auricular acupressure, pressure patches were replaced every 2 to 7 days, with some studies reporting replacement every 2 days (Yu, 2024; Yu et al., 2020), every 3 days (Yu et al., 2022; Chen et al., 2020; Chen et al., 2009; Wu et al., 2007), every 3–4 days (Ye and Zhan, 2023; Zhao et al., 2022), every 3–5 days (Chen, 2021), every 4 days (Wen et al., 2024), every 7 days (Liu et al., 2025), or every 2 weeks (Li J. et al., 2020). For auricular acupuncture, needles were typically retained for 24 to 72 h (Wang et al., 2024; Le et al., 2024), with alternating ears.

(5) Intervention cycle (total treatment duration)

Most studies employed 3–7 days per treatment course, with 2–4 courses totaling 2–4 weeks (Liu et al., 2025; Feng et al., 2025; Wang et al., 2024; Yu, 2024; Le et al., 2024; Xu, 2024; Lu, 2023; Ye and Zhan, 2023; Zhao et al., 2022; Yu et al., 2022; Chen, 2021; Chen et al., 2021; Wang, 2015; Ruan et al., 2014; Liu et al., 2014; Xu et al., 2013; Yang, 2012; Cai, 2011; Chen et al., 2009; Wu et al., 2007; Tang et al., 2005; Liu, 2004). Treatment cycles for some chronic PHN patients extended to 1 month (Wang et al., 2024; Chen, 2021; Yang, 2012) or longer (up to 60 days) (Zhao et al., 2024a).

In summary, significant heterogeneity exists in the stimulation parameters and intervention cycles across various auricular therapy modalities, with most studies lacking evidence-based medical rationale in protocol design.

#### 3.4.3 Reported outcome measures

The included studies employed a range of outcome measures to assess the effects of auricular therapy.

(1) Clinical efficacy: 29 studies assessed clinical efficacy by calculating the total effective rate (Liu et al., 2025; Feng et al., 2025; Wen et al., 2024; Wang et al., 2024; Zhao et al., 2024a; Yu, 2024; Le et al., 2024; Xu, 2024; Lu, 2023; Wang, 2023; Ye and Zhan, 2023; Zhao et al., 2022; Yu et al., 2022; Chen, 2021; Li J. et al., 2020; Yu et al., 2020; Wang et al., 2019; Wang, 2015; Ruan et al., 2014; Liu et al., 2014; Xu et al., 2013; Yang, 2012; Cai, 2011; Bao et al., 2010; Chen et al., 2009; Wu et al., 2007; Ding, 2007; Tang et al., 2005; Liu, 2004), primarily based on pain improvement and the “Diagnostic and Therapeutic Standards for Traditional Chinese Medicine Diseases.” Results suggested that auricular therapy may enhance treatment outcomes.
(2) Pain intensity: All studies assessed pain intensity in PHN patients (Liu et al., 2025; Feng et al., 2025; Wen et al., 2024; Wang et al., 2024; Zhao et al., 2024a; Yu, 2024; Le et al., 2024; Xu, 2024; Lu, 2023; Wang, 2023; Ye and Zhan, 2023; Zhao et al., 2022; Yu et al., 2022; Chen, 2021; Chen et al., 2021; Chen et al., 2020; Li J. et al., 2020; Yu et al., 2020; Wang et al., 2019; Wang, 2015; Ruan et al., 2014; Liu et al., 2014; Xu et al., 2013; Yang, 2012; Cai, 2011; Bao et al., 2010; Chen et al., 2009; Wu et al., 2007; Ding, 2007; Wang, 2007; Tang et al., 2005; Liu, 2004). 23 studies (Liu et al., 2025; Wen et al., 2024; Wang et al., 2024; Zhao et al., 2024a; Yu, 2024; Le et al., 2024; Xu, 2024; Lu, 2023; Ye and Zhan, 2023; Zhao et al., 2022; Chen, 2021; Li J. et al., 2020; Yu et al., 2020; Wang et al., 2019; Wang, 2015; Ruan et al., 2014; Liu et al., 2014; Xu et al., 2013; Cai, 2011; Bao et al., 2010; Chen et al., 2009; Ding, 2007; Wang, 2007) employed the Visual Analogue Scale (VAS), while four studies (Yang, 2012; Wu et al., 2007; Tang et al., 2005; Liu, 2004) did not specify pain assessment methods, one (Wang, 2023) employed subjective verbal ratings, while others (Liu et al., 2025; Wang, 2023; Yu et al., 2022; Chen et al., 2020; Yu et al., 2020) utilized more comprehensive tools such as the Numerical Rating Scale (NRS), McGill Pain Questionnaire (MPQ), and Short Form McGill Pain Questionnaire (SF-MPQ). All reported significant reduction in PHN pain intensity.
(3) Sleep quality (Liu et al., 2025; Wang et al., 2024; Zhao et al., 2024a; Le et al., 2024; Lu, 2023; Wang, 2023; Zhao et al., 2022; Yu et al., 2022; Chen et al., 2021; Yu et al., 2020; Ruan et al., 2014): Improvements in sleep disturbances among patients with postherpetic neuralgia (PHN) were primarily assessed using methods such as the Self-Reported Sleep Scale (SRSS) and the Pittsburgh Sleep Quality Index (PSQI). Results consistently reported significant reductions in sleep-related questionnaire scores compared to pre-treatment levels or control groups, suggesting that auricular therapy may improve sleep quality in PHN patients.
(4) Quality of life (Wang et al., 2024; Zhao et al., 2024a; Wang, 2023; Wang, 2007; Liu, 2004): Primarily assessed daily living quality in PHN patients, predominantly through the Short Form Health Survey (SF-36) and Traditional Chinese Medicine Quality of Life Evaluation Scale (CQ-11D) scores. Results reported significantly higher quality of life scores in the observation group or post-intervention patients compared to the control group or pre-enrollment levels, suggesting the intervention effectively improved patients’ quality of life.
(5) Objective biological indicators: Only 8 studies examined immune function and inflammation-related markers (Liu et al., 2025; Wen et al., 2024; Wang et al., 2024; Le et al., 2024; Xu, 2024; Yu et al., 2022; Chen et al., 2020; Li J. et al., 2020), such as serum C-reactive protein (CRP), interleukin-1β (IL-1β), interleukin-6 (IL-6), and tumor necrosis factor-*α* (TNF-α). All interventions reported reduced inflammatory responses in patients.
(6) Emotional and Psychological Status: 6 studies assessed emotional and psychological status using anxiety and depression scales (Wang et al., 2024; Le et al., 2024; Lu, 2023; Chen et al., 2021; Wang et al., 2019; Wang, 2007). Post-intervention, patients exhibited significant reductions in anxiety and depression scale scores, suggesting that the interventions effectively improved negative psychological emotions.
(7) Recurrence rate: 7 studies (Wen et al., 2024; Yu, 2024; Xu, 2024; Zhao et al., 2022; Chen, 2021; Xu et al., 2013; Chen et al., 2009) reported postoperative recurrence rates, with follow-up observations conducted 1–6 months or 1–2 years after intervention cessation. These findings consistently indicate that interventions significantly enhance treatment efficacy while reducing postoperative recurrence rates.
(8) Adverse reaction incidence: 13 studies (Liu et al., 2025; Feng et al., 2025; Yu, 2024; Le et al., 2024; Xu, 2024; Yu et al., 2022; Chen, 2021; Chen et al., 2020; Li J. et al., 2020; Yu et al., 2020; Wang, 2015; Liu et al., 2014; Yang, 2012; Bao et al., 2010; Chen et al., 2009; Wu et al., 2007; Ding, 2007; Wang, 2007; Tang et al., 2005) reported adverse reaction incidence to assess treatment safety, all indicating good patient tolerance to auricular therapy. Additionally, 7 studies (Wang et al., 2024; Le et al., 2024; Xu, 2024; Ye and Zhan, 2023; Wang et al., 2019; Yang, 2012; Ding, 2007) measured outcome indicators such as nursing satisfaction, Traditional Chinese Medicine symptom scores, time to analgesic onset, and therapeutic efficacy changes following auricular therapy interventions for PHN.

The results of the 32 included studies generally showed that the experimental group receiving auricular therapy combined with conventional treatment showed superiority over the control group or pre-intervention levels in reducing pain scores and improving the overall response rate, indicating that auricular therapy may have synergistic analgesic effects. Additionally, the findings suggest that auricular therapy can effectively alleviate insomnia symptoms in PHN patients, with a low incidence of adverse reactions and a good safety profile. A few studies also preliminarily confirmed the role of auricular therapy in regulating immune function and reducing inflammatory responses.

### 3.5 Potential mechanisms of action

Of the 32 included studies, 29 reported potential mechanisms of action for auricular therapy in treating PHN, whereas 3 did not explicitly discuss mechanisms. The reported mechanisms fell into three main categories: Traditional Chinese Medicine (TCM) theory, neurobiological regulation, and immune-inflammatory modulation.

**Figure 2:**
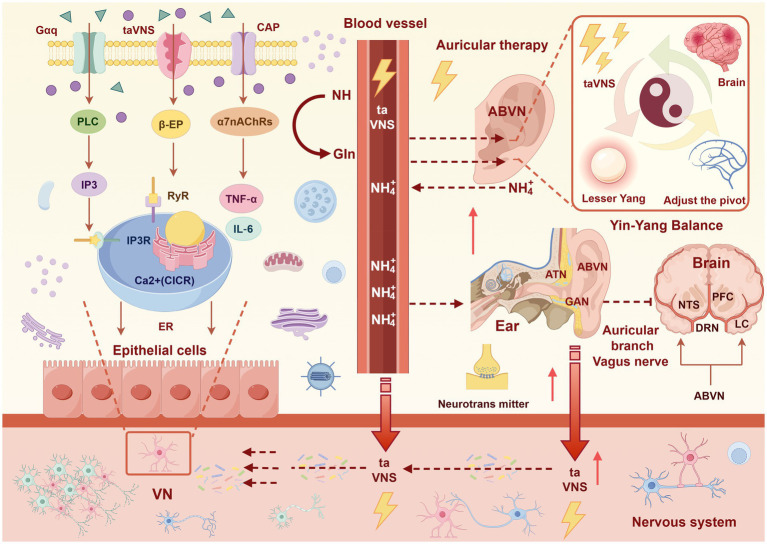
Possible mechanisms of auricular acupoint therapy for postherpetic neuralgia. This figure provides a visual synthesis of the mechanisms reported or implied in the 32 studies included in this systematic review (Liu et al., 2025; Feng et al., 2025; Wen et al., 2024; Wang et al., 2024; Zhao et al., 2024a; Yu, 2024; Le et al., 2024; Xu, 2024; Lu, 2023; Wang, 2023; Ye and Zhan, 2023; Zhao et al., 2022; Yu et al., 2022; Chen, 2021; Chen et al., 2021; Chen et al., 2020; Li J. et al., 2020; Yu et al., 2020; Wang et al., 2019; Wang, 2015; Ruan et al., 2014; Liu et al., 2014; Xu et al., 2013; Yang, 2012; Cai, 2011; Bao et al., 2010; Chen et al., 2009; Wu et al., 2007; Ding, 2007; Wang, 2007; Tang et al., 2005; Liu, 2004).

TCM-based mechanisms were most frequently cited (Feng et al., 2025; Zhao et al., 2024a; Yu, 2024; Xu, 2024; Lu, 2023; Ye and Zhan, 2023; Zhao et al., 2022; Yu et al., 2022; Chen, 2021; Chen et al., 2021; Li J. et al., 2020; Yu et al., 2020; Wang et al., 2019; Wang, 2015; Ruan et al., 2014; Xu et al., 2013; Yang, 2012; Cai, 2011; Bao et al., 2010; Tang et al., 2005), primarily involving “unblocking meridians, promoting qi and blood circulation, removing blood stasis, and relieving pain.” These mechanisms were commonly explained by the relationship between ear acupoints and zang-fu organs, as well as the holistic regulatory effects of auricular therapy. Neurobiological mechanisms were reported in 9 studies (Yu, 2024; Lu, 2023; Wang, 2023; Ye and Zhan, 2023; Li J. et al., 2020; Yu et al., 2020; Wang et al., 2019; Chen et al., 2009; Wang, 2007), including: (1) regulation of autonomic nervous system function (e.g., enhancing parasympathetic activity, reducing sympathetic tone); (2) modulation of cerebral cortex excitation and sedative-analgesic pathways; (3) promotion of endogenous analgesic substances (e.g., endorphin, enkephalin, serotonin, norepinephrine); and (4) inhibition of C-fiber afferent input and pain signal transmission through A-*δ* fiber activation. Immune-inflammatory mechanisms were reported in 10 studies (Liu et al., 2025; Wen et al., 2024; Wang et al., 2024; Le et al., 2024; Wang, 2023; Yu et al., 2022; Chen et al., 2020; Li J. et al., 2020; Yu et al., 2020; Chen et al., 2009), primarily involving: (1) suppression of pro-inflammatory cytokines (TNF-*α*, IL-1β, IL-6, IL-8, CRP); (2) regulation of T-lymphocyte subsets (increased Treg and Th2, decreased Th1 and Th17; (3) modulation of immunoglobulin levels (IgG, IgM); and (4) inhibition of CD4 + T-cell activation (reduced CD71 expression). One study on taVNS also measured α7 nicotinic acetylcholine receptor (α7nAChR) expression but found no significant change, leading the authors to suggest that other pathways may be involved (Chen et al., 2020). A visual synthesis of the proposed mechanisms is presented in Figure 2.

### 3.6 Summary of reported limitations

Only 8 of the 32 included studies explicitly discussed limitations. The most commonly reported limitations were short follow-up duration or lack of long-term efficacy assessment (Zhao et al., 2024a; Le et al., 2024; Yu et al., 2022; Chen et al., 2020; Chen et al., 2009), small sample size (Wang et al., 2024; Chen et al., 2020; Chen et al., 2009), and retrospective or single-center design (Wang et al., 2024; Wang, 2007). Two case reports (Chen et al., 2021; Wang, 2007) presented a low level of evidence. The remaining 24 studies did not report any limitations.

## 4 Discussion

This scoping review synthesizes evidence from 32 studies on auricular therapy for postherpetic neuralgia (PHN). The analysis delineates a range of applied modalities—including acupressure, acupuncture, bloodletting, and transcutaneous vagus nerve stimulation—and identifies consistent patterns in point selection, such as Shenmen, Subcortex, and Sympathetic. Reported outcomes primarily underscore significant improvements in pain intensity, sleep quality, and emotional distress, with a favorable safety profile. These collective findings affirm the therapeutic potential of auricular interventions. This is consistent with the results of previous studies (Luo et al., 2025; Wen et al., 2023). However, they also reveal substantial heterogeneity in protocols and a reliance on subjective measures, highlighting critical gaps that warrant further scrutiny in the following discussion of limitations and future directions.

### 4.1 Types of auricular therapy applied in PHN

Among the 32 studies included in this review, auricular acupressure is the most widely applied and frequently reported technique. The analgesic mechanism of auricular acupressure has been proposed to involve multiple effects (see Section 4.4 for details) (Wang, 2007). Bloodletting at the ear base and ear tip emphasizes “draining excess, removing stasis, and generating new tissue,” aligning with the TCM pathogenesis of PHN (Li B. et al., 2020). However, current studies on auricular bloodletting therapy remain limited in areas such as safety margins, blood volume assessment, infection risk management, and patient selection criteria. As an integration and extension of traditional Chinese medicine auricular acupuncture theory, traditional auricular acupuncture techniques, and modern neuroanatomy, the included literature indicates that transcutaneous auricular vagus nerve stimulation (taVNS) can reduce NRS pain scores in patients with postherpetic neuralgia (PHN), inhibit CD4 + T-cell activation, and reduce inflammatory cytokines (Chen et al., 2020). Several studies reported that combined approaches (e.g., auricular acupuncture with acupoint injection) improved both pain and quality of life (Zhao et al., 2024a). This provides that auricular therapy may serve as an adjunctive approach for pain management in PHN.

### 4.2 Intervention protocols: heterogeneity and the need for standardization

Although multiple studies have reported positive outcomes associated with auricular therapy for PHN, this review found significant heterogeneity among studies regarding acupoint selection, intervention frequency, stimulation duration, and total treatment duration. For instance, the replacement cycle for auricular patches ranged from 2 days to 2 weeks across studies (Liu et al., 2025; Wen et al., 2024), demonstrating substantial variation in this parameter. This heterogeneity may represent a primary obstacle to the clinical promotion and evidence-based practice of auricular therapy. The absence of unified standards or consensus is a common issue across various non-pharmacological intervention studies, suggesting that accelerating the development of standardized and regulated protocols may be crucial (Wang and Sun, 2022). Future efforts should focus on establishing standardized core protocols and exploring personalized, precision point selection strategies based on pain characteristics (e.g., burning pain, electric shock-like pain), Traditional Chinese Medicine syndromes (e.g., liver-gallbladder fire syndrome, qi stagnation and blood stasis syndrome), and neuropathological mechanisms. Clarifying the evidence-based rationale for core point selection and safety parameters or protocols will facilitate the integration of auricular therapy into multimodal pain management clinical pathways, particularly aiding its adoption in primary healthcare settings.

### 4.3 Reported outcome measures: from subjective reports to objective indicators

Current guidelines recommend commonly used PHN-related scales in clinical practice. These scales assess not only pain intensity but also pain characteristics, such as the ID Pain Screening Scale and the Diagnostic Questions on Pain (DN4) (Shen and Wang, 2024). Additionally, when evaluating PHN-related symptoms, quality of life assessments commonly employ scales such as the Short Form Health Survey-36 (SF-36) and the Nottingham Health Profile (NHP). The guidelines also strongly recommend psychological assessment tools like the Patient Health Questionnaire-9 (PHQ-9) and the Generalized Anxiety Disorder-7 (GAD-7) scale (National Pain Professional Quality Control Center Neuropathic Pain Expert Group, 2024). This study reveals that the outcome measures in the included studies primarily focused on subjective indicators such as patient-reported questionnaires or scales. While these measures are crucial for assessing patients’ subjective experiences, they are also susceptible to subjective factors and placebo effects, which to some extent limit the objectivity and comparability of the research findings. Notably, among the included studies, the application of immune-inflammatory markers (e.g., CRP, IL-6) showed a progressive increase from 2020 to 2025 (Liu et al., 2025; Wen et al., 2024; Wang et al., 2024; Le et al., 2024; Xu, 2024; Yu et al., 2022; Chen et al., 2020; Li J. et al., 2020). For example, Chen et al. (2020) reported that taVNS reduced pain intensity in patients with drug-refractory PHN and also measured inflammatory markers. Yu et al. (2022) reported reductions in serum CRP, IgG, and IgM levels following auricular bloodletting combined with acupoint pressure therapy. Results showed that serum C-reactive protein (CRP), immunoglobulin G (IgG), and immunoglobulin M (IgM) levels in the intervention group decreased significantly compared to pre-treatment levels (*p* < 0.05), with a more pronounced reduction than in the control group. Furthermore, only seven studies evaluated recurrence rates through follow-up assessments. Existing research has been conducted over relatively short periods, primarily focusing on short-term efficacy. Consequently, there remains insufficient evaluation of the long-term outcomes of auricular therapy in managing PHN, including its effectiveness, advantages, and recurrence rates.

### 4.4 Potential mechanisms of action

The analgesic effects of auricular therapy in PHN are likely mediated by multiple interconnected pathways, including peripheral-central neural input, descending inhibition, endogenous opioid release, immune-inflammatory modulation, autonomic nervous system regulation, and emotion-sleep interactions (Chen et al., 2023). However, direct mechanistic evidence remains limited, and most proposed pathways are derived from preclinical studies or indirect clinical measurements rather than definitive human neurobiological data.

Traditional Chinese Medicine perspective. From a TCM perspective, PHN pathogenesis involves two interrelated processes:

(1) Accumulation of fire toxins and damp-heat obstructing meridians, leading to impaired qi and blood circulation (“pain due to obstruction”);
(2) Constitutional deficiency or prolonged illness depleting qi and blood, resulting in failure to nourish tendons and vessels (“pain due to deficiency of nourishment”) (Zhang et al., 2023). Auricular therapy is hypothesized to counteract these processes by unblocking meridians, promoting qi circulation, and replenishing blood. Specific acupoints are selected based on their purported functions: Shenmen and Heart for sedation and analgesia; Liver for pain relief and stagnation dispersal (Liu et al., 2022); and Spleen, as the “root of acquired constitution,” for replenishing qi and blood to address deficiency-related pain (Zhao et al., 2024b). Filiform needle acupuncture at auricular points is thought to unblock qi and blood in the skin region and harmonize yin and yang (Li et al., 2024). While this framework has guided clinical practice for centuries, it remains largely untested by modern mechanistic methods.

Modern neurobiological perspective. From a contemporary neurobiological standpoint, several candidate mechanisms have been proposed. Auricular acupressure has been associated with autonomic nervous system modulation (reduced sympathetic tone, enhanced parasympathetic activity), suppression of pro-inflammatory cytokines (IL-6, TNF-α, CRP), improved local microcirculation, and reduced neural edema (Li M. C. et al., 2025; Jiang et al., 2022). Bloodletting therapy has been reported to improve microcirculation, downregulate inflammatory factors, enhance local oxygen supply, and modulate immune activation, potentially accelerating neuroinflammation resolution and tissue repair (Wei and Huang, 2022). For taVNS, emerging evidence suggests that it may inhibit neuroinflammation and suppress peripheral immune activation, although the precise molecular targets remain incompletely defined (Chen et al., 2020). Notably, the study by Chen et al. (2020) incorporated the α7 nicotinic acetylcholine receptor (α7 nAChR) as an indicator. Results revealed no significant change in its expression compared to pre-treatment levels, suggesting that the mechanism by which taVNS exerts its effects on PHN may involve other pathways or factors. This finding is inconsistent with previous research (Chen et al., 2023) and represents a new avenue worthy of further exploration.

### 4.5 Limitations and future research directions

In addition to the research limitations summarized in Section 3.5, this scoping review has the following limitations: First, the majority of included studies were conducted in Chinese, with only one English-language study, and research over the past five years has been predominantly focused on China. This may be attributed to the fact that most ear-related projects conducted abroad have centered on auricular acupuncture and transcutaneous vagus nerve stimulation (tVNS), while international scholars have directed greater efforts toward developing shingles vaccines (Ecarnot and Michel, 2022; Oh et al., 2024). Second, the included original studies themselves exhibited inconsistent methodological quality, commonly characterized by small sample sizes and inadequate reporting of randomization and blinding implementation, which limits the interpretability of the findings. Current mechanistic validation largely relies on short-term inflammatory markers or small-sample studies, lacking systematic support from neuroimaging, neuroelectrophysiology, and animal experiments. Furthermore, as a scoping review, this study did not conduct a systematic methodological quality assessment of the included studies, which is consistent with the methodological framework of scoping reviews.

Based on the above analysis, future research directions may consider the following points. (1) Conduct multicenter, large-sample, rigorously designed randomized controlled trials to enhance the level of evidence and credibility. (2) Focus on in-depth research to validate objective mechanisms. Integrate modern neuroscience and molecular biology techniques, incorporating objective biomarkers and neurophysiological or imaging indicators to explore, at functional and molecular levels, the key pathways regulated by auricular therapy—particularly emerging modern techniques like transcutaneous vagus nerve stimulation (taVNS)—in managing PHN neuropathic pain. (3) Develop and validate standardized intervention protocols. Determine optimal combinations of auricular points, stimulation frequencies, and treatment durations for different auricular therapies, and disseminate these through clinical practice guidelines. (4) Evaluate long-term efficacy by monitoring extended follow-up and recurrence rates to assess the value and advantages of auricular therapy in the chronic management of PHN.

## 5 Conclusion

This scoping review summarized existing evidence from 32 publications on auricular therapy for postherpetic neuralgia. Multiple auricular interventions—including auricular acupressure, auricular acupuncture, bloodletting therapy, auricular massage, and transcutaneous auricular vagal nerve stimulation (taVNS)—were reported to alleviate pain and improve associated symptoms such as sleep disturbances and emotional distress, with a favorable safety profile as reported in the included studies. However, this field remains constrained by significant heterogeneity in treatment protocols, reliance on subjective outcome measures, and a lack of long-term efficacy data. Future efforts should focus on precise point selection, objective outcome measures, individualized treatment strategies, and mechanistic investigations. Establishing unified clinical guidelines may help optimize the use of auricular therapy in long-term PHN care and pain management.

## Data Availability

The original contributions presented in the study are included in the article/supplementary material, further inquiries can be directed to the corresponding author.
